# Factors Associated with Healthcare Utilization in Children with Sickle Cell Disease in Saudi Arabia

**DOI:** 10.3390/ijerph23030309

**Published:** 2026-03-01

**Authors:** Daniya Sabrah, Seyed M. Karimi, Bert Little, Demetra Antimisiaris, Danyah A. Aldailami, Ahmed Alabdrabalnabi, Fatima Aldarweesh

**Affiliations:** 1 Department of Health Management and Systems Sciences, School of Public Health, University of Louisville, Louisville, KY 40202, USA; seyed.karimi@louisville.edu (S.M.K.); bert.little@louisville.edu (B.L.); demetra.antimisiaris@louisville.edu (D.A.); fatima.aldarweesh@louisville.edu (F.A.); 2 Department of Public Health, College of Health Sciences, Saudi Electronic University, Riyadh 93499, Saudi Arabia; d.aldailami@seu.edu.sa (D.A.A.); a.alabdrabalnabi@seu.edu.sa (A.A.)

**Keywords:** sickle cell disease, healthcare utilization, pediatric, Saudi Arabia, retrospective study, Andersen model, regional disparities, comorbidity

## Abstract

**Highlights:**

**Public health relevance—How does this work relate to a public health issue?**

- This study addresses the substantial and costly burden that sickle cell disease (SCD) places on healthcare systems, a significant public health challenge in Saudi Arabia.
- It investigates patterns of healthcare resource consumption—inpatient, outpatient, and emergency care—highlighting system pressures from a major chronic pediatric condition.

**Public health significance—Why is this work of significance to public health?**

- It identifies modifiable clinical factors (e.g., complication count and crisis episodes) and treatment choices (e.g., hydroxyurea and transplant) that drive healthcare utilization, pointing to potential targets for intervention.
- It reveals significant regional disparities in service use within Saudi Arabia, underscoring inequities in healthcare access, delivery, or disease management that require public health attention.

**Public health implications—What are the key implications or messages for practitioners, policymakers, and/or researchers?**

- For policymakers and health system planners: Findings call for targeted resource allocation and strategies to mitigate regional disparities and support for preventive therapies (e.g., hydroxyurea) and curative options (e.g., transplant) that may reduce high-acuity visits over time.
- For clinicians and researchers: Results emphasize the importance of proactive complication management and systematic severity assessment (e.g., using comorbidity indices) in routine care to potentially prevent hospitalizations and optimize outpatient follow-up.

**Abstract:**

(1) Background: In Saudi Arabia, a high-income country with a publicly funded healthcare system, sickle cell disease (SCD) remains a major pediatric health challenge. This study aimed to identify factors associated with healthcare utilization, specifically inpatient (IP), outpatient (OP), and emergency department (ED) visits, among children with SCD in Saudi Arabia. (2) Methods: A retrospective observational study was conducted using data from the KAIMRC registry (2015-2023), including 450 children under 12 years old diagnosed with SCD. Negative binomial regression models were employed to analyze the annual average visits, accounting for clinical, demographic, and regional healthcare resource variables. (3) Results: Key predictors of IP visits included complication count, crisis episodes, and region (eastern, western, and southern regions had higher utilization than central). ED visits were positively associated with complications, crisis episodes, and hydroxyurea use, but negatively associated with bone marrow transplant receipt. OP visits increased with higher Charlson Comorbidity Index scores, age, and bone marrow transplant, but were lower in the eastern region. (4) Conclusions: These findings highlight the influence of clinical and regional factors even within an equitable, high-resource healthcare system.

## 1. Introduction

Sickle cell disease (SCD)-related hospitalizations are mainly due to vaso-occlusive crisis (VOC) (i.e., painful crisis) [1] or due to common complications that are associated with SCD (i.e., hemolytic anemia and organ system complications). Healthcare utilization and cost patterns are significantly higher for patients diagnosed with SCD-related chronic conditions or complications [2]. Largely due to hospital readmission rates, the healthcare utilization costs in the U.S. are about $2.4 billion per year for patients diagnosed with SCD [3]. The type of healthcare utilization varies across patients and time and may depend on patient-related factors. While patients with SCD, compared to other disease or condition patients, have worse health outcomes, it is partly due to access to fewer health resources [4].

The higher healthcare costs associated with SCD are primarily due to reliance on acute care services rather than primary care utilization [2]. Higher healthcare utilization rates are also associated with VOCs [5]. Understanding the factors associated with emergency department visits among such patients can help better address healthcare needs [6] and help reduce overall healthcare cost burdens.

Moreover, the Centers for Disease Control (CDC) recognized the geographic location and outpatient healthcare utilization pattern as a research priority among patients diagnosed with SCD [7].

In Saudi Arabia, the prevalence rate of SCD is 2.6%, with about 49.6% prevalence affecting the children population mainly because of consanguineous marriages [8,9]. It costs about U.S. $395 million annually in Saudi Arabia to manage SCD for patients diagnosed with it [10], largely driven by frequent hospitalizations, emergency department visits, and the management of complications such as vaso-occlusive crises (VOCs). Factors like parental education and medication compliance tend to affect the frequency of acute care visits compared to outpatient diagnosis (OPD) and missed OPD appointments [9].

Limited literature explores factors affecting the type of healthcare utilization among the Saudi Arabia population [9,11]. However, the existing studies only look at emergency visits and conduct correlational analysis. The studies do not account for potential confounders like Charlson Comorbidity Scores (CCI), seasonality, healthcare resources, and all Saudi regions. This is the first study to utilize the Andersen and Aday health services research model [12] to identify factors affecting healthcare utilization among Saudi Arabia children diagnosed with SCD. Based on searches of literature databases, Google, grey literature via connectedpapers.com, and manual bibliography reviews of key relevant publications, our study is the first to determine factors affecting all types of healthcare utilization (i.e., inpatient, outpatient, and emergency visits) among children diagnosed with SCD in Saudi Arabia. Our study is also the first to account for CCI, the total number of crisis episodes, VOCs, the total number of complications, bone marrow procedure receipt, regional health resources factors (i.e., the total number of medical doctors, nurses, and beds in hospitals) and include data from all Saudi Arabia regions. The Andersen and Aday behavioral model of health services utilization provides a structured framework to examine predisposing, enabling, and need factors. Its application to SCD in Saudi Arabia allows for a systematic exploration of both patient- and system-level determinants, including regional resources, a particularly relevant dimension in a geographically diverse country with universal health coverage. Therefore, this study aims to identify factors influencing healthcare utilization in Saudi Arabia using the Andersen and Aday health services research model and the King Abdullah International Medical Research Center (KAIMRC) database that includes all Saudi Arabia regions.

## 2. Materials and Methods

### 2.1. Study Design, Population, and Sample

This study is a retrospective observational study. Patient records were identified by using the International Classification of Diseases (ICD-10) diagnoses codes for sickle cell disorders (D57), specifically those for sickle cell crises (D57.0) and sickle cell without crises (D57.1) diagnoses that appear in the hospital’s electronic records. Sickle cell disease as a principal diagnosis from 2015 to 2023 was used to identify patients for study inclusion. The study included children up to 11 years old who were diagnosed with SCD between the study period 2015–2023 in Saudi Arabia. We excluded children above 11 years as per the definition by the Centers for Disease Control (CDC) milestone development, which considers children above 11 years as teenagers [13] or children with incomplete data. Based on the inclusion criteria mentioned, 450 patients were included as the final study sample.

### 2.2. Data Source

The de-identified KAIMRC patient data was extracted for the study. The MNG-HA data provided by KAIMRC provides care from primary to tertiary to all National Guard soldiers, their dependents, and individuals residing in Saudi Arabia [14]. The MNG-HA system primarily serves National Guard employees, their dependents, and eligible Saudi citizens within its catchment areas. In emergency situations, care may also be provided to non-citizens and uninsured individuals, though the majority of the pediatric SCD cohort in this study are Saudi nationals affiliated with the National Guard. The ethics committee of the University of Louisville approved this study (24.0655), and KAIMRC received data access approval with approval number IRB/1452/24. Because of this study’s retrospective, observational design, no informed consent was needed.

KAIMRC is a multicenter registry database with several hospitals serving the Ministry of National Guard beneficiaries. Some of the hospitals included are King Abdulaziz Medical City (KAMC-R) in Riyadh, the largest site representing the central region, and King Abdulaziz Medical City (KAMC-J) in Jeddah, representing the western region. The registry also covers smaller hospitals such as King Abdulaziz Hospital in Al Ahsa, Al-Imam Abdulrahman bin Faisal Hospital in Dammam, serving the eastern region, and Prince Mohammad bin Abdulaziz Hospital in Al Madinah [15]. Additionally, we utilized the Ministry of Health Saudi Arabia’s publicly available health resource information to merge it with our secondary [16].

### 2.3. Outcome Variable

The study outcome variable is the average annual number of inpatient (IP), outpatient (OP), and emergency department (ED) visits, and it is the count variable. The variable values were calculated by dividing the total number of IP, OP, and EP visits for each unique patient by the total number of days a patient was in the data within the study period 2015–2023, multiplied by 365. The variables represent the healthcare utilization outcome construct of the Andersen and Aday model [12].

### 2.4. Independent Variables

The study utilized the Andersen and Aday health services research model [12] to inform the study data analysis variable selection process. The enabling factors construct variables included are health resource information (i.e., the total number of physicians, nurses, hospital beds, physicians per 100 beds, and nurses per 100 physicians in all of Saudi Arabia), a numeric variable for each year of the study period. The predisposing factors variables are age, a continuous variable, and gender (i.e., male and female), a categorical variable. The geographical variable, region, is categorical and identifies the central, east, west, and south regions. The need factor construct variables are patient clinical characteristics (i.e., CCI scores, the number of complications, and the number of crisis episodes), which are continuous variables. The severity of SCD was clinically measured through variables (i.e., CCI score, number of complications, and number of crisis episodes). The treatment variables are categorical (i.e., bone marrow procedure variable values, Yes and No receipt, hydroxyurea medication variable values, Yes and No). The ICD-10 version CCI scores were assigned to each patient based on a widely utilized CCI scoring system that assesses severity based on 19 conditions, as our data included only ICD-10 codes [17]. The Charlson Comorbidity Index (CCI) was applied using ICD-10 codes as per the method [17]. This is a generic comorbidity measure, not SCD-specific, but it provides a standardized estimate of comorbid burden. Complications counted included both acute SCD-related events (e.g., acute chest syndrome, stroke, and priapism) and chronic organ dysfunctions (e.g., renal disease and retinopathy), as recorded in the KAIMRC registry.

After the initial pre-regression analysis, we excluded hospital beds, physicians per 100 beds, and nurses per 100 physicians, as these variables posed a multicollinearity problem. The total number of nurses and physicians was summed as a single variable and multiplied by 10,000, as the number varied significantly by 10,000 every other year throughout the study period. We used the total number of physicians and nurses per region as a proxy for overall healthcare capacity. While pediatric or hematology-specific staffing data would be ideal, they were not consistently available across all regions and years. Total workforce metrics correlate with broader healthcare infrastructure and accessibility, which indirectly influence pediatric SCD care.

### 2.5. Statistical Analysis and Empirical Model

Data analysis was conducted using STATA SE 18 for three regression models. The linear regression for continuous and Poisson and negative binomial regression for count data as our outcome variable is count in nature. By running both adjusted linear and count data models, sensitivity assessment of our coefficients was performed, comparing across all models. The count data models were employed for both marginal effects and incidence rate ratios (IRR). The pre-regression test analysis included omitted variable bias and multicollinearity tests. Additionally, we performed bivariate analysis to identify estimates of each independent variable. The empirical model of our analysis is described below:

$$\begin{array}{cc} Y_{i}=exp(\beta_{0}\;+ & \beta_{1}Age_{i}+\beta_{2}CrisisEpisodes_{i}+\beta_{3}Complications_{i}+\beta_{4}Gender_{i}\\ & +\beta_{5}Region_{i}+\beta_{6}BoneMarrowTreatment_{i}\\ & +\beta_{7}HydroxyUrea_{i}+\beta_{8}TotalNursesPhysicians_{i}+\beta_{9}CCI_{i}) \end{array}$$

where i indicates an individual and Y denotes the study outcome variable (i.e., the average annual number of IP, OP, or ED hospital visits) separately for three regression models. CrisisEpisodes represents the total number of crisis episodes, Age is the age of patients in data, and Complications is the total number of complications for each patient in data. Gender is a categorical variable with two values (i.e., male or female). Region is central, east, west, and south. BoneMarrowTreatment is a categorical variable if bone marrow transplant treatment is received, which takes the value Yes or No. Similarly, HydroxyUrea is a categorical variable if the patient received hydroxyurea medication and takes the value of Yes or No. TotalNursesPhysicians and CCI are numeric variables, where TotalNursesPhysicians is the total number of nurses and physicians in the patient’s region of residence. At the same time, CCI is the total CCI score of each patient in the data that takes a value of 0 or ≥1.

Model fit was assessed using likelihood ratio tests. The negative binomial model was selected over linear and Poisson regression due to overdispersion in the count data. Detailed model comparisons are provided in Table S1 of the Supplementary Materials.

## 3. Results

### 3.1. Study Variables and Hospital Visit

Our study sample included 450 patients < 12 years of age diagnosed with SCD: 450 utilized IP care, 330 utilized ED, and 449 utilized OP hospital visits (Table 1). The patient characteristics did not differ significantly across the three types of hospital visits. The mean age of the sample utilizing the average annual number of IP hospital visits was 5 years (SD = 3.2 years), the total number of complications was 0.3 (SD = 0.5), the total number of crisis episodes was 3.9 (SD = 7.0), and the total number of physicians and nurses was 41,390 (SD = 20,675). The mean age of the study sample utilizing the average annual number of ED hospital visits was 4.6 years (SD = 3.1), the total number of complications was 0.3 (SD = 0.5), the total number of crisis episodes was 5.3 (SD = 7.7), and the total number of physicians and nurses was 38,217 (SD = 19,923). Similarly, the sample characteristics did not differ for OP visit type, with a mean age of 5 years (SD = 3.2), the total number of complications was 0.3 (SD = 0.5), the total number of crisis episodes was 3.9 (SD = 7.0), and the total number of physicians and nurses was 41,390 (SD = 20,698).

### 3.2. Treatment Variables

The patient characteristics differed slightly for treatment variables (i.e., bone marrow receipt and hydroxyurea medication) among ED visit types (Table 2). A total of 20.4% of patients utilizing IP visits received bone marrow treatment, 5.3% had ≥1 CCI score, 70.9% received hydroxyurea, and 44.7% were female; 46.4% resided in central, 30.9% in east, and 22.7% in west and south regions of Saudi Arabia, respectively. A total of 13.3% of patients utilizing ED visits received bone marrow treatment, 4.5% had ≥ CCI score, 78.2% received hydroxyurea, and 44.5% were female; 42.1% resided in central, 36.4% in east, and 21.5% in west and south regions of Saudi Arabia, respectively. A total of 20.5% of patients utilizing OP visits received bone marrow treatment, 5.3% had ≥1 CCI score, 71% received hydroxyurea, and 44.5% were female; 46.5% resided in central, 31% in east, and 22.5% in west and south regions of Saudi Arabia, respectively.

### 3.3. Average Annual Number of Inpatient Hospital Visits

The IP visit results are not sensitive to the methods we chose for modeling, as described in Table S2 of the Supplementary Materials. However, since our outcome variable is count data, we will interpret both the marginal effects and IRR for the negative binomial model. The average annual IP visit was higher by 0.37 units (95% CI = 0.06–0.69) with an increase in each additional complication, 0.06 units (95% CI = 0.04–0.08) for those with CCI score ≥1 compared to those with 0, 5.04 units (95% CI = 3.37–6.71) with 10,000 increase in total number of nurses and physicians, 1.83 units (95% CI = 0.86–2.81) for those residing in east Saudi Arabia region compared to central, and 1.09 units (95% CI = 0.59–1.60) for those residing in the west and south Saudi Arabia region compared to central region, respectively.

The IRR estimated by the negative binomial model for the total number of complications is 23% (1.23, 95% CI = 1.03–1.47), which indicates a 23% increase in IP visits with a higher number of complications. The IP visit was higher by 3% (1.03, 95% CI = 1.02–1.05) with a 1-unit increase in the annual number of crisis episodes. There is a 100% (2.65, 95% CI = 1.68–4.17) increase in IP visits for those residing in eastern Saudi Arabia than those in central. Similarly, there is a 98% (1.98, 95% CI = 1.44–2.71) increase in IP visits for those residing in the west and south regions compared to the central region of Saudi Arabia. There was a statistically significant effect of total nurses and physicians increase. However, it was very small in terms of effect estimates, as shown in Table S2, Supplementary Materials.

### 3.4. Average Annual Number of Emergency Hospital Visits

The ED visit results are not sensitive to the methods we chose for modeling. However, since our outcome variable is count data, we will interpret both the marginal effects and IRR for the negative binomial model. The average annual ED visit was higher by 0.37 (95% CI = 0.16–0.58) units with an increase in each additional complication, 0.04 units (95% CI = 0.02–0.05) with an increase in each additional crisis episode, 2.21 units (95% CI = 1.04–3.38) with 10,000 increases in total number of nurses and physicians, and 1.35 units (95% CI = 0.55–2.15) for those residing in east Saudi Arabia region compared to central. There were higher ED visits by 0.48 units (95% CI = 0.21–0.75) for those who received hydroxyurea compared to those who did not. In contrast, those who received bone marrow transplants had a decrease in ED visits by 0.97 units (95% CI = −1.31, −0.63) compared to those who did not.

The IRR estimated by the negative binomial model for the total number of complications is a 52% (1.52, 95% CI = 1.20–1.94) increase in ED visits with higher number of complications. The ED visit increased by 4% (1.04, 95% CI = 1.03–1.06) with higher crisis episodes. There is a 100% (3.41, 95% CI = 1.86–6.26) increase in ED visits for those who reside in the eastern Saudi Arabia region compared to those in central. There is a 72% (1.72, 95% CI = 1.26–2.36) increase in ED visits for those using hydroxyurea compared to those without. In contrast, there is a decrease in ED visits by 67% (0.33, 95% CI = 0.22–0.49) for those who received bone marrow transplants compared to those who did not. There was a statistically significant effect of total nurses and physicians increase. However, it was very small in terms of effect estimates, as shown in Table S3 of Supplementary Materials.

### 3.5. Outpatient Hospital Visit

**Figure 1:**
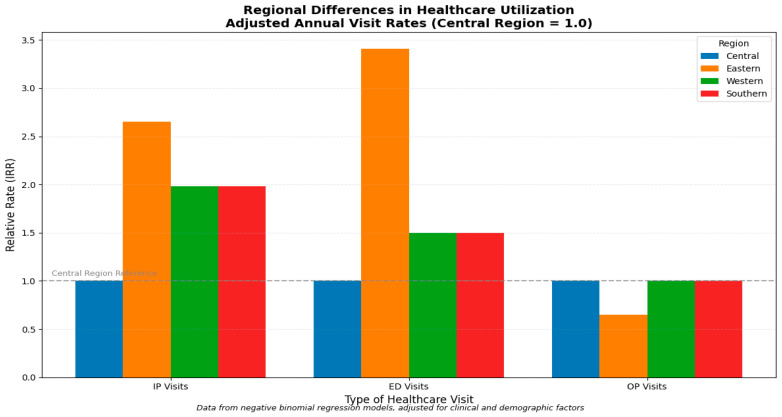
Regional differences in healthcare utilization.

The OP visit results are not sensitive to the methods we chose for modeling, as described in Table S4 of Supplementary Materials. However, since our outcome variable is count data, we will interpret both the marginal effects and IRR for the negative binomial model. The average annual OP visit increases by 2.12 units (0.51–3.74) for those with CCI score ≥1 compared to those with 0, 0.13 units (95% CI = 0.01–0.25) with each year increase in age, 4.41 units (95% CI = 3.26–5.56) for those who received bone marrow transplant compared to those who did not. In contrast, those residing in the east Saudi Arabia region had decreased OP visits by 2.86 units (95% CI = −4.50, −1.22) compared to those residing in central Saudi Arabia as shown in Figure 1. 

The IRR estimated by the negative binomial model for CCI score is a 33% (1.33, 95% CI = 1.07–1.66) increase in OP visits for those with a score ≥ 1 compared to those with 0. The OP visits increase by 2% (1.02, 95% CI = 1.00–1.03) with an increase in each year of age. There is an 81% (1.81, 95% CI = 1.56–2.12) increase in OP visits for those who received bone marrow transplants compared to those who did not. In contrast, there is a decrease in ED visits by 35% (0.65, 95% CI = 0.51–0.84) for those who reside in the east region of Saudi Arabia compared to those in the central region, as shown in Table S4 of the Supplementary Materials.

## 4. Discussion

The present study identified key factors that affect the type of hospital visit, confirming the results with existing studies within and outside Saudi Arabia. The important predictors of the average annual number of IP hospital visits are the total number of complications, the total number of crisis episodes [18], total healthcare resources in a region (i.e., the total number of nurses and physicians), and the patient’s region of residence in Saudi Arabia. The total number of crisis episodes and complications in the eastern, western, and southern Saudi Arabia regions, compared to the central ones, is positively associated with the type of IP hospital. Similarly, the important predictors of the average annual number of ED hospital visit type are total number of complications, total number of crisis episodes, bone marrow treatment receipt, hydroxyurea medication receipt, total number of nurses and physicians, and Saudi Arabia region compared to the central region [19,20]. Bone marrow receipt is negatively associated with ED hospital visits [21]. It aligns with the literature, which claims that painful episodes are often alleviated after a bone marrow transplant, resulting in reduced ED department visits [22,23].

In contrast, the total number of complications; crisis episodes [6,24]; hydroxyurea medication receipt, probably due to increased toxicity [25]; and east region compared to central, probably due to difference in patient demographics [19], are positively associated with the average annual number of ED hospital visit types. In our study, hydroxyurea use was associated with higher ED visits. The positive association between hydroxyurea use and ED visits likely reflects confounding by indication: children with more severe disease and higher healthcare utilization are more likely to be prescribed hydroxyurea. Thus, hydroxyurea may be a marker of disease severity rather than a driver of ED visits. It is important to note that hydroxyurea remains a cornerstone of SCD management and is associated with reduced long-term complications and mortality. The observed association should not be interpreted as a negative effect of hydroxyurea, but rather as a marker of disease severity in this cohort. However, our study is the first to identify regional differences by accounting for all regions of Saudi Arabia in the statistical modeling for each hospital visit type. Finally, the important predictors of the average annual number of OP hospital visit types are the CCI score, receipt of bone marrow treatment, age, and the eastern region of Saudi Arabia compared to the central region. The receipt of bone marrow treatment, CCI score, and age are positively associated with OP receipt, which is explained by the need for follow-up OP visits after receiving bone marrow for monitoring purposes or management of comorbidities, as per standard post-transplantation guidelines [26]. In contrast, the east region is negatively associated with OP visit type compared to the central region.

### 4.1. Study Limitations

The study accounts for unaccounted factors missing in existing relevant studies (e.g., CCI score, Saudi Arabia regions, and healthcare resource information). However, it does not account for unobserved confounders such as genotype, race/ethnicity, family income, parental education, health literacy, barriers to healthcare access, and seasonality due to data limitations. Therefore, the study does not necessarily provide conclusive evidence of causation. The unaccounted confounder barriers to healthcare access variables may not affect the study estimates in the Saudi Arabian context, as the healthcare system in Saudi Arabia is similar to a universal healthcare system, where people can choose between free public insurance or private insurance, regardless of their income level. Additionally, our study did not differentiate between SCD genotypes (e.g., HbSS, HbSC, and HbSβ-thalassemia), which are known to vary in clinical severity and thus may influence healthcare utilization differently. Regional variations in complications may also reflect genetic differences, such as fetal hemoglobin (HbF) levels, which are known to vary across Saudi regions and influence stroke risk and disease severity. Future studies should integrate genetic data to explore these underlying biological factors.

The study acknowledges validity threats, such as generalizability, as the present study results are limited to National Guard employees and their family members. However, for ED visits, irrespective of their military status, patients admitted with injury without insurance are treated. Additionally, it acknowledges the presence of selection bias due to unobserved confounders within the study context. If parental income and education level are positively associated with the number of IP hospital visits and negatively associated with the total number of crisis episodes, then the lack of information on parental income and education level would lead to an underestimation of the association between the number of hospital IP visits and the number of crisis episodes. In contrast, if parental income and education level are positively associated with regional and healthcare resource estimates, omitting these variables from the statistical model due to a lack of information might lead to an overestimation of the association between the number of nurses and physicians and Saudi Arabia’s regions. However, the effect might not be significantly different because, as mentioned previously, the healthcare system is free to access regardless of parental income or insurance status. Furthermore, the study lacked data on socioeconomic determinants such as parental income, education, insurance type, and health literacy, which are known to influence healthcare utilization. Although Saudi Arabia’s healthcare system provides broad coverage, subtle disparities related to socioeconomic status may still exist and were not captured in this analysis. Future studies should aim to incorporate such variables to better understand equity in SCD care.

The psychosocial status of patients (i.e., depression, anxiety, and social support) is negatively associated with IP utilization adherence and bone marrow treatment receipt; it might lead to an overestimation of the association between bone marrow receipt and IP visits. In contrast, if hospital policies and health infrastructure are positively associated with IP visits and the total number of nurses, physicians, and regions, the lack of information on this would lead to an overestimation of the association between the total nurses, physicians, and IP visits.

While the study population is drawn from the MNG-HA system, which may not fully represent all socioeconomic or geographic subgroups in Saudi Arabia, the structure of healthcare delivery and access under this system is broadly consistent with the public healthcare framework across the Kingdom. However, caution should be exercised in generalizing these findings to all Saudi children with SCD, particularly those in purely private or non-integrated care settings. Furthermore, our measure of healthcare resources was limited to quantitative data (number of physicians and nurses). We did not capture qualitative aspects such as staff training, adherence to clinical guidelines, availability of specialized equipment (e.g., automated exchange transfusion devices), or institutional protocols, which may also influence utilization patterns. Future studies should aim to incorporate such quality metrics where feasible.

Suppose the parental income and education level are negatively associated with ED visit type and the total number of crisis episodes. In that case, the lack of information will lead to overestimating the association between ED visits and the number of crisis episodes. In contrast, if parental income and education level are positively associated with hydroxyurea adherence in Saudi Arabia, the lack of information would lead to an underestimation of the association between ED visits and hydroxyurea or other regions. If hospital-level infrastructure, like ED admission policies and bed availability, are positively associated with ED visit types and healthcare resources, such as the number of nurses, physicians, and regions of Saudi Arabia, a lack of information would lead to an overestimation of association between ED visit type and health resource factors like total nurses and physicians.

If parental income is positively associated with OP visits (due to transportation barriers) and negatively associated with CCI scores, the lack of information on parental income would lead to underestimation of the association between OP visits and CCI scores. However, the effect would not be significant compared to present estimates, as Saudi healthcare is free for all. If the psychosocial status of patients (i.e., depression, anxiety, and social support) is negatively associated with OP visit type and bone marrow treatment receipt, the lack of information on it will lead to an overestimation of the association between OP visit and bone marrow treatment receipt. Lastly, if hospital infrastructure, such as telemedicine or other appointment availability and capacity types, is positively associated with OP visits and healthcare resources like the total number of nurses and physicians, as well as in Saudi Arabia regions, the lack of information on it would lead to overestimation of the association between OP visits and healthcare resources or Saudi Arabia regions.

### 4.2. Policy Implications

The present study results help inform Saudi Arabia’s policies for preventive care, as improving OP and preventive care infrastructure can help reduce ED department visits for patients with higher CCI scores and the total number of complications. Region-specific interventions, such as differences in ED department use among patients residing in the east and southwest, compared to central, could be due to underlying disparities in regional healthcare access (i.e., imbalance in primary care and specialist availability). Patients with higher CCI scores were associated with IP visits, increasing healthcare costs. Identifying high-risk patients, providing them with necessary preventive healthcare resources to mitigate IP visits, and improving parent health literacy could help reduce healthcare cost burdens. The CCI, designed for adults, may not fully capture pediatric SCD morbidity. Most comorbidities in this cohort were stroke-related. Future studies might benefit from using a pediatric-modified comorbidity index or SCD-specific severity scores.

Our results inform the association between comorbidity and complications with healthcare utilization. So in the SCD population, a rigorous disease management and care coordination might be developed to facilitate early interventions and improve healthcare utilization burden. The implementation could include relevant accessible healthcare services to manage comorbidities or complications by either allowing healthcare services to reach patients’ homes in certain circumstances or providing patients with transportation facilities or resources in case they cannot visit a hospital or miss the visit.

## 5. Conclusions

The study provided evidence of factors that affect the type of hospital visits (i.e., IP, OP, and ED departments). The factors that affect the type of healthcare utilization are patient demographics, clinical characteristics, and regional healthcare resource factors for the entire Kingdom of Saudi Arabia. Future studies can build on the study results to inform close to true causal relationships for factors that predict the type of healthcare utilization by accounting for identified unobserved confounders.

## Figures and Tables

**Table 1:** Descriptive statistics of numeric study variables by type of hospital visit.

Variable	Inpatient (N = 450)		Emergency (N = 330)		Outpatient (N = 449)	
	Mean	SD	Mean	SD	Mean	SD
Total # of complications	0.25	0.48	0.29	0.51	0.25	0.48
Total # of crisis episodes	3.91	6.99	5.26	7.72	3.92	6.99
Age	4.99	3.21	4.60	3.08	5.00	3.20
Total # of nurses and physicians	41,391	20,675	38,217	19,923	41,390	20,698

**Table 2:** Descriptive statistics of categorical study variables by type of hospital visit.

		Type of Visit					
		Inpatient		Emergency		Outpatient	
		N = 450		N = 330		N = 449	
		Frequency	Percentage	Frequency	Percentage	Frequency	Percentage
Bone marrow treatment reciept							
	Yes	92	20.44	44	13.33	92	20.49
	No	358	79.56	286	86.67	357	79.51
CCI score							
	0	426	94.67	315	95.45	425	94.65
	≥1	24	5.33	15	4.55	24	5.35
Hydroxyurea medication reciept							
	Yes	319	70.89	258	78.18	319	71.05
	No	131	29.11	72	21.82	130	28.95
Female							
	0	249	55.33	183	55.45	249	55.46
	1	201	44.67	147	44.55	200	44.54
Region							
	Central	209	46.44	139	42.12	209	46.55
	East	139	30.89	120	36.36	139	30.96
	West and South	102	22.67	71	21.52	101	22.49

## Data Availability

The study data can be made available upon reasonable request and approval of Saudi Arabia KAIMRC ethics approval to view or access the data.

## Supplementary Materials

The following supporting information can be downloaded at: https://www.mdpi.com/article/10.3390/ijerph23030309/s1, Table S1: Model Comparison by each Type of Hospital Visit, Table S2: Factors Affecting an Average Annual Number of Inpatient Hospital Visits with Estimates across Multiple Regression Models and the Respective 95% CI, Table S3: Factors Affecting an Average Annual Number of Emergency Hospital Visits with Estimates across Multiple Regression Models and respective 95% CI, Table S4: Factors Affecting an Average Annual Number of Outpatient Hospital Visits with Estimates across Multiple Regression Models and respective 95% CI.
