# Supplementary material for: Factors Associated with Healthcare Utilization in Children with Sickle Cell Disease in Saudi Arabia
Source: Int J Environ Res Public Health. 2026 Mar 1;23(3):309. doi: 10.3390/ijerph23030309 (PMC13027078; doi:10.3390/ijerph23030309)
Supplement: Supplementary file 1 [file ijerph-23-00309-s001.zip › ijerph-4076201-supplementary.pdf]

*Supplementary Material*

**Table S1.** Model Comparison by each Type of Hospital Visit.

| | Model | |
|---|---|---|
| | Poisson | Negative Binomial |
| Model assumption | No overdispersion parameter | Overdispersion parameter |
| Average annual number of Inpatient hospital visits | | |
| Estimated alpha value (CI) | | 0.22** (0.10-0.46) |
| Log pseudo-likelihood | -777.60 | -763.93 |
| Average annual number of Emergency hospital visits | | |
| Estimated alpha value (CI) | | 0.46**(0.21-1.00) |
| Log pseudo-likelihood | -618.08 | -592.45 |
| Average annual number of Outpatient hospital visits | | |
| Estimated alpha value (CI) | | 0.17**(0.13-0.23) |
| Log pseudo-likelihood | -1390.15 | -1235.96 |

**Table S2.** Factors Affecting an Average Annual Number of Inpatient Hospital Visits with Estimates across Multiple Regression Models and the Respective 95% Confidence Intervals.

| ***Inpatient visit*** | **Marginal effects (95% CI)** | | | **Incidence Rate Ratio (95% CI)** | |
|---|---|---|---|---|---|
| | **Linear** | **Poisson** | **Negative Binomial** | **Poisson** | **Negative Binomial** |
| CCI score | 0.54 | 0.45* | 0.38 | 1.28* | 1.24 |
| | (-0.27,1.34) | (-0.05,0.94) | (-0.22,0.98) | (0.97,1.69) | (0.88,1.75) |
| Total # of complications | 0.38* | 0.37*** | 0.37** | 1.23*** | 1.23** |
| | (-0.02,0.77) | (0.11,0.62) | (0.06,0.69) | (1.06,1.42) | (1.03,1.47) |
| Total # of crisis episode | 0.07*** | 0.05*** | 0.06*** | 1.03*** | 1.03*** |
| | (0.04,0.10) | (0.04,0.07) | (0.04,0.08) | (1.02,1.04) | (1.02,1.05) |
| Bone marrow treatment reciept | -0.20 | -0.20 | -0.09 | 0.89 | 0.95 |
| | (-0.75,0.36) | (-0.57,0.17) | (-0.53,0.34) | (0.73,1.10) | (0.74,1.21) |
| Hydroxyurea medication reciept | -0.02 | 0.05 | 0.01 | 1.03 | 1 |
| | (-0.48,0.44) | (-0.28,0.37) | (-0.38,0.39) | (0.86,1.23) | (0.80,1.25) |
| Total # of nurses and physicians | 4.78*** | 4.93*** | 5.04*** | 1.00*** | 1.00*** |
| | (2.87,6.69) | (3.47,6.38) | (3.37,6.71) | (1.00,1.00) | (1.00,1.00) |
| Age | -0.03 | -0.03 | -0.03 | 0.98 | 0.98 |
| | (-0.08,0.03) | (-0.07,0.01) | (-0.08,0.01) | (0.96,1.01) | (0.96,1.01) |
| Female | 0.11 | 0.08 | 0.05 | 1.04 | 1.03 |
| | (-0.25,0.46) | (-0.19,0.32) | (-0.24,0.34) | (0.91,1.19) | (0.87,1.22) |
| Reference: Central region | | | | | |
| East region | 1.59*** | 1.90*** | 1.83*** | 2.67*** | 2.65*** |
| | (0.73,2.46) | (1.03,2.76) | (0.86,2.81) | (1.80,3.98) | (1.68,4.17) |
| West and South region | 1.12*** | 1.09*** | 1.09*** | 1.96*** | 1.98*** |
| | (0.49,1.75) | (0.65,1.52) | (0.59,1.60) | (1.49,2.57) | (1.44,2.71) |
| **N** | | | **450** | | |

p value significance ***1%, **5%, *10%

**Table S3.** Factors Affecting an Average Annual Number of Emergency Hospital Visits with Estimates across Multiple Regression Models and respective 95% Confidence Intervals.

| ***Emergency Visit*** | **Marginal effects (95% CI)** | | | **Incidence Rate Ratio (95% CI)** | |
|---|---|---|---|---|---|
| | **Linear** | **Poisson** | **Negative Binomial** | **Poisson** | **Negative Binomial** |
| CCI score | -0.56* | -0.58** | -0.45 | 0.53** | 0.60 |
| | (-1.20,0.08) | (-1.07,-0.09) | (-0.99,0.09) | (0.31,0.91) | (0.32,1.11) |
| Total # of complications | 0.47*** | 0.38*** | 0.37*** | 1.51*** | 1.52*** |
| | (0.15,0.78) | (0.21,0.54) | (0.16,0.58) | (1.26,1.81) | (1.20,1.94) |
| Total # of crisis episode | 0.06*** | 0.03*** | 0.04*** | 1.03*** | 1.04*** |
| | (0.04,0.09) | (0.02,0.04) | (0.02,0.05) | (1.02,1.05) | (1.03,1.06) |
| Bone marrow treatment reciept | -0.95*** | -1.04*** | -0.97*** | 0.32*** | 0.33*** |
| | (-1.38, -0.51) | (-1.34,-0.74) | (-1.31,-0.63) | (0.23,0.46) | (0.22,0.49) |
| Hydroxyurea medication reciept | 0.49*** | 0.52*** | 0.48*** | 1.76*** | 1.72*** |
| | (0.12,0.85) | (0.29,0.75) | (0.21,0.75) | (1.37,2.26) | (1.26,2.36) |
| Total # of nurses and physicians | 2.77*** | 2.41*** | 2.21*** | 1.00*** | 1.00*** |
| | (1.25,4.29) | (1.43,3.39) | (1.04,3.38) | (1.00,1.00) | (1.00,1.00) |
| Age | 0.01 | 0.01 | 0.00 | 1.01 | 1 |
| | (-0.03,0.06) | (-0.02,0.03) | (-0.03,0.03) | (0.98,1.04) | (0.97,1.04) |
| Female | 0.08 | 0.04 | 0.08 | 1.04 | 1.08 |
| | (-0.21,0.36) | (-0.13,0.20) | (-0.13,0.28) | (0.87,1.24) | (0.86,1.37) |
| Reference: Central region | | | | | |
| East region | 1.33*** | 1.43*** | 1.35*** | 3.47*** | 3.41*** |
| | (0.63,2.02) | (0.77, 2.10) | (0.55,2.15) | (2.13,5.64) | (1.86,6.26) |
| West and South region | 0.34 | 0.23* | 0.20 | 1.39* | 1.36 |
| | (-0.16,0.84) | (-0.02,0.47) | (-0.09,4.93) | (0.97,1.97) | (0.87,2.12) |
| **N** | | | **330** | | |

p value significance ***<1%, **<5%, *<10%

**Table S4.** Factors Affecting an Average Annual Number of Outpatient Hospital Visits with Estimates across Multiple Regression Models and respective 95% Confidence Intervals.

| *Outpatient Visit* | **Marginal effects (95% CI)** | | | **Incidence Rate Ratio (95% CI)** | |
|---|---|---|---|---|---|
| | **Linear** | **Poisson** | **Negative Binomial** | **Poisson** | **Negative Binomial** |
| CCI score | 2.63** | 1.78*** | 2.12** | 1.27*** | 1.33** |
| | (0.35,4.92) | (0.85,2.72) | (0.51,3.74) | (1.12,1.44) | (1.07,1.66) |
| Total # of complications | 0.51 | 0.47* | 0.4 | 1.07* | 1.05 |
| | (-0.61,1.63) | (-0.04,0.99) | (-0.43,1.23) | (1.00,1.14) | (0.94,1.18) |
| Total # of crisis episode | 0.03 | 0.04* | 0.04 | 1.00* | 1.01 |
| | (-0.05,0.11) | (-0.00,0.08) | (-0.02,0.10) | (1.00,1.01) | (1.00,1.01) |
| Bone marrow treatment reciept | 6.14*** | 4.17*** | 4.41*** | 1.76*** | 1.81*** |
| | (4.58,7.70) | (3.50,4.83) | (3.26,5.56) | (1.60,1.92) | (1.56,2.12) |
| Hydroxyurea medication reciept | -0.04 | -0.11 | -0.19 | 0.96 | 0.97 |
| | (-1.34,1.27) | (-0.79,0.58) | (-1.22,0.83) | (0.90,1.08) | (0.85,1.12) |
| Total # of nurses and physicians | 4.06 | 3.37*** | 2.91 | 1*** | 1 |
| | (-1.36,9.48) | (-9.13,5.83) | (-1.03,6.85) | (1.00,1.00) | (1.00,1.00) |
| Age | 0.20** | 0.19*** | 0.13** | 1.03*** | 1.02** |
| | (0.04,0.37) | (0.11,0.27) | (0.01,0.25) | (1.01,1.04) | (1.00,1.03) |
| Female | 0.02 | 0.05 | -0.13 | 1.01 | 0.98 |
| | (-0.99,1.02) | (-0.44,0.53) | (-0.89,0.64) | (0.94,1.07) | (0.89,1.09) |
| Reference: Central region | | | | | |
| East region | -2.07* | -2.82*** | -2.86*** | 0.66*** | 0.65*** |
| | (-4.54,0.39) | (-3.91, -1.75) | (-4.50, -1.22) | (0.56,0.78) | (0.51,0.84) |
| West and South region | 0.71 | 0.72 | 0.93 | 1.09 | 1.11 |
| | (-1.07,2.50) | (-0.24,1.69) | (-0.61,2.49) | (0.97,1.22) | (0.93,1.33) |
| **N** | | | **449** | | |

p value significance ***<1%, **<5%, *<10%
